# Occupational exposure to organophosphorus and carbamates in farmers in La Cienega, Jalisco, Mexico: oxidative stress and membrane fluidity markers

**DOI:** 10.1186/s12995-020-00283-y

**Published:** 2020-10-28

**Authors:** Joel Salazar-Flores, Fermín P. Pacheco-Moisés, Genaro G. Ortiz, Juan H. Torres-Jasso, Odette Romero-Rentería, Ana L. Briones-Torres, Erandis D. Torres-Sánchez

**Affiliations:** 1 Department of Medical Sciences and Life, CUCIENEGA, University of Guadalajara, Ocotlan, Jalisco Mexico; 2 Department of Chemistry, CUCEI, University of Guadalajara, Guadalajara, Jalisco Mexico; 3 Department of Philosophical and Methodological Discipline, CUCS, University of Guadalajara, Guadalajara, Jalisco Mexico; 4 Department of Biological Sciences, CUCOSTA, University of Guadalajara, Puerto Vallarta, Jalisco Mexico

**Keywords:** Oxidative stress, Organophosphorus, Carbamates, Occupational exposure, Farmers of Cienega, Membrane fluidity

## Abstract

**Background:**

The region of La Cienega in Jalisco Mexico, is an important agricultural reference for the production of corn, sorghum and wheat, among other grains, so the use of pesticides for pest control is high. However, in this rural area there are no toxicological studies that assess the occupational risk of pesticide use. Therefore, this study is the first to determine the oxidative stress levels markers (GSH, GSSG, carbonyl groups, nitric oxide metabolites and lipid peroxides) as well as alteration of the mitochondrial membrane fluidity caused by occupational exposure to organophosphorus and carbamates in farmers of this region. This occupational risk can increase cellular oxidation, which explains the high prevalence of neurodegenerative diseases and cancer in Cienega settlers to be analyzed in future studies.

**Methods:**

Comparative cross-sectional study was performed using two groups: one not exposed group (*n* = 93) and another one with occupational exposure (*n* = 113). The latter group was sub-divided into 4 groups based on duration of use/exposure to pesticides. Oxidative stress levels and membrane fluidity were assessed using spectrophotometric methods. Statistical analyses were performed using SPSS software ver. 19.0 for windows.

**Results:**

The most commonly used pesticides were organophosphorus, carbamates, herbicide-type glyphosate and paraquat, with an average occupational exposure time of 35.3 years. There were statistically significant differences in markers of oxidative stress between exposed farmers and not exposed group (*p* = 0.000). However, in most cases, no significant differences were found in markers of oxidative stress among the 4 exposure sub-groups (*p* > 0.05).

**Conclusion:**

In the Cienega region, despite the indiscriminate use of organophosphorus and carbamates, there are no previous studies of levels oxidative stress. The results show increased levels of oxidative stress in occupationally exposed farmers, particularly membrane fluidity levels increased three times in contrast to not exposed group.

## Introduction

Oxidative stress is an imbalance between reactive oxidant species (ROS) generation and antioxidant species, which results in oxidation of biomolecules [1]. Oxidizing species lose electrons and generate molecules with unpaired electrons called free radicals, which react with other molecules through redox reactions [1]. When the levels of oxidants exceed the levels of antioxidant species, biomolecules become oxidized, resulting in various chronic diseases, neurodegenerative diseases and cancer [2]. Physiologically, the balance between oxidation and antioxidation is mediated by the enzyme glutathione reductase (GR) which converts oxidized glutathione (GSSG) to reduced glutathione (GSH). The GSH is a substrate for glutathione peroxidase (GPx), an enzyme that reduces oxidant species such as hydroperoxides [3]. The organophosphorus compounds (OPs) alter the antioxidant defense and biomembranes lipids, resulting in mitochondrial energy depletion, proteolysis and DNA fragmentation [4]. OPs are highly lipid-soluble, volatile and toxic to non-target organisms, including humans [5]. Some OPs like terbufo are chemically unstable thioether OPs with unpaired electrons [6]. It destabilizes protein and lipid molecules [7, 8]. The carbofuran is a carbamate pesticide and carbamic acid derivative which stimulates cholinergic hyperactivity and modifies redox potential while decreasing antioxidant status [9]. Consequently, factors that trigger systemic toxicity to pesticides are ROS and reactive nitrogen species (RNS) [9–11]. The ROS are formed by partial reduction of molecular oxygen, and the main products are superoxide anion (O_2_^−^) and H_2_O_2_ [12–14]. RNS are molecules derived from the chemical reaction between nitric oxide (NO) and O_2_^−^ to forms peroxynitrite (ONOO^−^) releasing nitrite (NO_2_^−^), nitrate (NO_3_^−^) and OH^−^ groups [15].

Increased in levels of ROS and RNS affect the side groups of proteins and form carbonyl groups. The carbonyl groups react particularly with lysine, cysteine and histidine [16]. This may impact on cysteine groups of GR, thereby interfering with the conversion of GSSG to GSH [3]. Another potential impact of oxidation as a result of occupational exposure to pesticides is reflected in peroxidation of polyunsaturated fatty acids (PUFAs), particularly arachidonic acid, to form products such as malondialdehyde (MDA) and 4-hydroxyalkene (4-HNE) [17]. Increases in products of lipid peroxidation alter the membrane lipids, thereby affecting biophysical parameters such as fluidity, permeability, domains formation, fission-fusion, cellular signals transduction and activities of membrane proteins [18, 19], which can alter the level of membrane fluidity important in cellular and mitochondrial integrity. It should be noted that in la Cienega region of Jalisco México, there are no studies that evaluate organophosphorus toxicological damage at membrane fluidity levels. By other side, it is known that ONOO^−^ oxidizes lipids faster than ROS, and it forms peroxynitrosocarbonates which increase the harmful effects mediated by pesticides [20, 21]. In particular, high occupational exposure to OPs inhibits the enzyme acetylcholinesterase (AChE), leading to over-stimulation of the cholinergic activity and the glutamatergic pathway. Increased glutamate activates N-methyl-D-aspartate (NMDA) receptors which, which in turn stimulate the synthesis of NO [21, 22]. Moreover, the OPs increase the concentration of intracellular Ca^2+^ ions, further inducing NO synthesis [22]. The toxicity of carbamate is regulated by a process similar to that of OPs through irreversible inhibition of AChE, with increased oxidative stress. In the study by Liu [23], it was observed that exposure of liver cells to carbamate induced the synthesis of cytotoxic aldehydes (MDA and 4-HNE), acrolein and H_2_O_2_. In addition, the toxicity of paraquat increases the concentration of H_2_O_2_ and OH^−^ radicals, and H_2_O_2_ promotes the formation of disulphide bonds, thereby reducing antioxidant capacity [24].

The aim of this study was to evaluate the indices of oxidative stress (GSH, GSSG, carbonyl groups, NO metabolites and lipid peroxides) and membrane fluidity in farmers with occupational exposure to pesticides, relative to not exposed group without occupational exposure in La Cienega region, Jalisco, Mexico. This region is characterized by the lack of regulation on the sale and application of pesticides [25] and by the absence of toxicological studies that impact on health, so the analysis of oxidative stress levels is a first approach to address the health problems that affect The Cienega region in Jalisco, Mexico. The most widely used pesticides were terbufos (s-tert-butylthiomethyl-o, o-diethyl-phosphorodithioate), carbofurans (2,3-dihydro-2,2-dimethyl-7-benzofuranyl methyl carbamate),paraquat (1,1′-dimethyl-4,4′-bipyridyl dichloride), glyphosate [n- (phosphonomethyl) glycine-isopropylamine 1: 1), and fipronil (5-amino-1- (2,6-dichloro-α, α, α -trifluoro-p-tolyl) -4- trifluoromethylsulfinylprazole-3-carbonitrile)] [26, 27].

## Material and methods

### Study sample

A total of 113 residents of La Cienega region of Jalisco, Mexico, occupationally exposed to various pesticides, were studied. The average occupational exposure time was 35.3 years, and the subjects were aged 22 to 72 years. Sampling was carried out from 2017 to 2018 in the corn growing months (high exposure period) in 7 agricultural communities of La Cienega region, Jalisco, Mexico. The not exposed group was made up of 93 subjects with an age range between 17 to 28 years and a mean of 23 years, with a 95% confidence interval, without occupational exposure to pesticides, who are residents of the same geographical area. This project was approved by the Ethics Committee of the University Center of La Cienega, University of Guadalajara (Folio 2017–037). Each participant signed an informed consent letter guaranteeing the confidentiality of data. The study was carried out in strict compliance with the principles of the Declaration of Helsinki.

### Processing of the samples

Blood (10 ml) was taken through venous puncture in two 5-mL vials. One of the vials had 0.1% ethylene diamine tetra acetic acid (EDTA). Plasma, serum and erythrocytes were obtained after centrifugation at 310 g for 15 min at 4 °C, and kept at − 80 °C until used.

### Determination of oxidative stress markers and MF

#### Glutathione redox system

Erythrocyte samples were divided in two fraction for total and oxidized glutathione quantification using an enzymatic recycling procedure. For total glutathione determination, GSH was oxidized to GSSG with 5,5′-dithiobis-2-nitrobenzoic acid. Subsequently, GSSG was reduced to GSH in a reaction catalyzed by the enzyme GR, with Nicotinamide Adenine Dinucleotide Phosphate (NADPH) as reductant. The reaction rate of 5,5′-dithiobis-2-nitrobenzene was determined at 412 nm. The other fraction of the samples was treated as above, except that 2-vinylpyridine was added to remove all GSH. The GSSG levels were subtracted from the total glutathione to determine the GSH level [28, 29].

#### Protein carbonyl levels and metabolites of nitric oxide

Plasma (200 μL) was vortexed with 500 μL of 10 mM 2,4-dinitrophenylhydrazine diluted in 2 M HCl. Subsequently, it was incubated for 1 h at room temperature. Thereafter, 333 μL of trichloroacetic acid was added, followed by centrifugation at 14,000 rpm for 20 min. The precipitate was washed three times with 1 mL of ethanol: ethyl acetate solution (1:1, v: v). To the final precipitate was added 600 μL of guanidine hydrochloride, followed by incubation for 15 min at room temperature. The absorbance of the solution was read at 370 nm [30].

Nitric oxide metabolites were determined by adding 6 mg of zinc sulfate to 400 μL of serum, and then centrifuging at 10,000 rpm at 4 °C for 10 min. To the resultant supernatant was added 100 μL of vanadium chloride at a concentration of 8 mg/mL. To reduce the NO_3_^−^ to NO_2_^−^, Griess reagent (comprising 50 μL of 2% sulfanilamide, and 50 μL of 0.1% N-(1-naphthyl) ethylenediamine dihydrochloride) was added. Following incubation for 30 min at 37 °C, the absorbance was read at 540 nm [31].

#### Lipid peroxide levels and membrane fluidity

The quantification of MDA and 4-HNE products in plasma was performed using FR12 kits (Oxford Biomedical Research, MI, USA) in line with the manufacturer’s protocol. In this assay, the reagent N-methyl-2-phenylindole reacts with MDA and 4-HNE at 45 °C to form a chromophore which is determined via absorbance measurement at 586 nm (Oxford kit).

The fluidity of the plasma membranes was determined in platelets via the incorporation of 1,3 dipyrylpropane (DiPP). In this method, 1 mL of sample was added to 0.1 nmol of DiPP in 10 mM Tris-HCl buffer, pH 7.8. The mixture was incubated in the dark at 4 °C for 5 h to ensure incorporation of DiPP. Then, fluorescence was measured at 24 °C at an excitation wavelength of 329 nm for the monomer and the excimer; and the emission peaks were read at 379 and 480 nm, respectively. Finally, the excimer/monomer fluorescence ratio of the samples was measured [32].

### Statistical analysis

To calculate the frequency of use of pesticides and values of biomarkers of oxidative stress, descriptive statistics were performed. The results are expressed as frequencies and mean ± standard deviation, respectively. Student’s *t*-test was used to determine significant differences in the values of biomarkers of oxidative stress among the different groups (exposed and unexposed), while ANOVA and Tukey multi-comparative tests were used to compare exposure groups based on the duration of use of pesticides. All statistical analyses were carried out using SPSS Statistical Program v. 19.0 [33]. For each marker, statistical significant of difference was assumed at *p* ≤ 0.05.

## Results

### The most frequently used pesticides in La Cienega region

The pesticides most used by farmers were terbufos (18.7%), carbofuran (21.4%), fipronil (8.2%), and alphacypermethrin (3.6%). The other pesticides had lower percentages of exposure, relative to total OPs which had 23.39% frequency (Table 1). It is noteworthy that some herbicides also had high frequency of use. These were paraquat (19.02%) and glyphosate (8.5%).

**Table 1:** Frequency of use pesticides in La Cienega de Jalisco México region, and classification

Pesticide	n (%)	Type	US EPA	WHO
Malathion	1 (0.30)	Organophosphorus	D	II
Parathion	1 (0.30)	Organophosphorus	C	1ª IA
Diazinon	2 (0.61)	Organophosphorus	E	II
Chlorpyrifos-ethyl	11 (3.37)	Organophosphorus	C	II
Terbufos	61 (18.7)	Organophosphorus	E	IA
**Percentage of Organophosphorus = 23.39%**				
Cypermethrin	3 (0.92)	Pyrethroid	C	II
Tefluthrin	6 (1.84)	Pyrethroid	NL	1B
Deltamethrin	2 (0.61)	Pyrethroid	NL	II
Cyhalothrin-lambda	4 (1.22)	Pyrethroid	D	II
Cypermethrin-alpha	12 (3.6)	Pyrethroid	C	II
**Percentage of pyrethroids = 8.19%**				
Carbofuran	70 (21.4)	Carbamate	NL	1B
Paraquat	62 (19.02)	Bipyridil	E	II
Glyphosate	28 (8.5)	Aminophosphonate	E	III
Fipronil	27 (8.2)	Phenylpyrazole	NL	II
Glufosinate-ammonium	18 (5.52)	Phosphinates	C	1B
Propaquizafop	6 (1.84)	Aryloxyphenoxypropionate	NL	U
Lindane	2 (0.61)	Organochlorine	NL	II
Imidacloprid	5 (1.53)	Neonicotinoid	E	II
Atrazine	2 (0.61)	Triazine	NL	III
Chlorantraniliprole	1 (0.30)	Anthraminic diamides	NL	U
Acetochlor	1 (0.30)	Chloroacetanilides	B2	III
Fenbutatin oxide	1 (0.30)	Organotin	E	III

U.S. EPA (Environmental Protection Agency)- Carcinogenecity Categorization: *B2,* Probable human carcinogen; *C,* Possible human carcinogen; *D,* Not classifiable as to human carcinogenicity; *E,* Evidence of not carcinogenicity for humans. *NL,* Not likely to be carcinogenic to humans. WHO (World Health Organization)- classification of pesticides by hazard: *IA,* Extremely hazardous; *IIB*, Highly hazardous; *II,* Moderately hazardous; *III,* Slightly hazardous; *U,* Unlikely to present acute hazardous effect

### Usage of the major pesticides and occupational exposure time

**Fig. 1:**
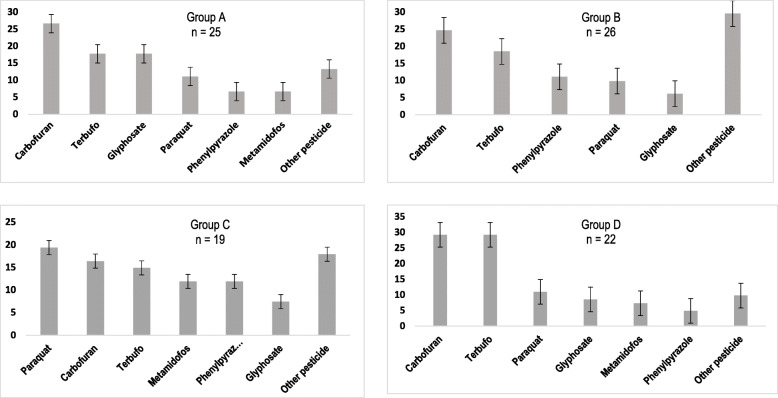
Time of exposure (in years) of pesticides in The Cienega, Jalisco, Mexico area. For the 4 different sub-groups of subjects, the most frequently used pesticides were OPs and carbamates

After determining biomarkers of oxidative stress, the occupationally exposed group was divided into four subgroups based on the duration of exposure to pesticides: sub-group A comprised farmers who used pesticides up to 10 years (mean age 32 years); sub-group B farmers used pesticides up to 20 years (mean age 46 years); sub-group C farmers used pesticides up to 30 years (mean age 53 years), while farmers in sub-group D were those who used pesticides for more than 30 years (mean age 62 years) (see Fig. 1). These subgroups show a higher percentage of men exposed in contrast to women (Table 2). In addition, this grouping confirmed that in the four sub-groups, there was high usage of pesticides, as was evident in long durations of exposure to terbufos (15 to 29 years), carbofuran (18 to 28 years), glyphosate (6 to 18 years), and paraquat (10 to 18 years). Although the farmers used other pesticides including phenylpyrazoles, the most commonly used ones were OPs and carbamates (Fig. 1).

**Table 2:** Percentage of men and women in occupationally exposed subjects and not-exposed subjects

	Unexposed Group (%) ***n*** = 93	Group A (%) ***n*** = 25	Group B (%) ***n*** = 26	Group C (%) ***n*** = 19	Group D (***%***) ***n*** = 22
**Male**	53.25	53.57	76	53.85	91.18
**Female**	46.75	46.43	24	46.15	8.82

### Oxidative stress markers in farmers and not-exposed subject

There were statistically significant differences in values of oxidative stress markers between occupationally exposed and unexposed subjects, as shown in Table 3.

**Table 3:** Oxidative stress markers in occupationally exposed subjects and not-exposed subjects

Markers	Subjects occupationally exposed to pesticides (***n*** = 113)	Subjects occupationally not exposed to pesticides (***n*** = 93)	***P*** value*
GSH (μM)	0.0024 ± 0.00155	1.58 ± 0.608	0.0000
GSSG (μM)	0.0011 ± 0.00339	0.5221 ± 0.5900	0.0000
GSH/GSSG ratio	2.18 ± 0.4572	3.0262 ± 1.0305	0.581
Carbonyl groups in proteins (μmol/mL)	62.7 ± 35.70	13.57 ± 9.04	0.0000
Nitrates-Nitrites (μmol/mL)	55.62 ± 55.26	5.06 ± 1.81	0.0000
Lipoperoxides (MDA-4HNE; μmol/mL)	4.82 ± 2.00	1.59 ± 0.88	0.0000
Membrane fluidity	0.49 ± 0.03	0.14 ± 0.05	0.0000

Data are presented as mean values ± standard deviation

*Value obtained with the t-Student test for independent samples

In order to investigate whether the duration of use of pesticides affected oxidative stress biomarkers, average values of each oxidative stress marker in the not-exposed group was compared among the 4 exposure sub-groups (A, B, C and D). In all cases except glutathione redox system, the unexposed group showed lower oxidative stress values than any of the 4 sub-groups (Table 4). The same effect was observed on comparing markers between the exposed and unexposed groups (Table 3). Subsequent statistical analysis with ANOVA revealed that the 5 groups (one unexposed and 4 exposed) differed significantly except GSH/GSSG (Table 4).

**Table 4:** Oxidative stress markers in not-exposed subjects and occupationally exposed group

	Unexposed Group (***n*** = 93)	Group A (***n*** = 25)	Group B (***n*** = 26)	Group C (***n*** = 19)	Group D (***n*** = 22)	P-Value*
**GSH (μmol/mL)**	1.58 ± 0.6080	0.0027 ± 0.0019	0.0023 ± 0.0086	0.0026 ± 0.0017	0.0026 ± 0.0018	(*F* = 126.20, *p* = 0.000)
**GSSG (μmol/mL)**	0.5221 ± 0.5900	0.0007 ± 0.0004	0.0009 ± 0.0034	0.0006 ± 0.0003	0.0009 ± 0.0005	(*F* = 13.84, *p* = 0.000)
**GSH/GSSG**	3.026 ± 1.0305	3.857 ± 3.75	2.555 ± 2.52	4.333 ± 4.66	2.888 ± 2.6	(*F* = 0.582, *p* = 0.710)
**Carbonyl groups in proteins (μmol/mL)**	13.57 ± 9.3	49.44 ± 25.62	80.05 ± 47.77	47.51 ± 15.95	71.06 ± 41.44	(*F* = 48.63, *p* = 0.000)
**Nitrates-Nitrites (μmol/mL)**	5.06 ± 1.8	59.06 ± 55.33	54.49 ± 42.83	63.01 ± 75.09	45.17 ± 28.0	(*F* = 22.78, *p* = 0.000)
**Lipoperoxides MDA-4HNE (μmol/mL)**	1.59 ± 0.89	4.94 ± 1.90	4.22 ± 1.23	4.85 ± 1.96	5.80 ± 2.16	(*F* = 65.57, *p* = 0.000)
**Membrane fluidity**	0.14 ± 0.04	0.49 ± 0.03	0.49 ± 0.04	0.47 ± 0.02	0.49 ± 0.05	(*F* = 694.84, *p* = 0.000)

Data are presented as mean values ± standard deviation

*Value obtained with the one-way ANOVA. The comparison was made between the unexposed group and subgroups A, B, C and D, for each oxidative stress marker

### Effect of pesticide exposure on glutathione redox system

There were significant differences in GSH and GSSG levels between the not exposed group and the 4 exposed sub-groups (*p* = 0.000). This may be attributed to the not-exposure of the second group, since when sub-group A was compared with sub-group B, C or D, there were no significant differences (*p* = 0.963, *p* = 0.980 and *p* = 1.000 respectively); nor were there any significant differences when sub-group B was compared against sub-group C (*p* = 1.000), B against D (*p* = 0.950), or sub-group C against D (*p* = 0.970). The *p* values for GSSG levels have the similar statistical effect: there were differences only with the not exposed group, as shown in Table 4. GSH/GSSG ratio was not significant differences between the not exposed and the 4 exposed sub-groups (*p* = 0.710) as shown in Table 4.

**Fig. 2:**
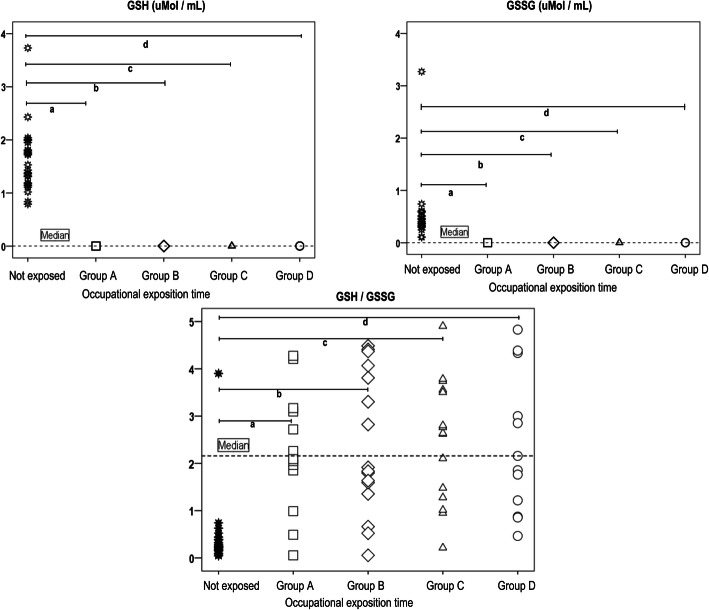
GSH (μMol / mL) and GSSG (μMol / mL) levels and GSH/GSSG ratio in groups of occupational exposure and not exposed group. GSH and GSSG results for subgroups A, B, C, D shown nearly values in the scale for which it is observed as a single point in the graph. *p* values were obtained with a one-way ANOVA test at 95% confidence level. The significance value is shown for the comparison of the unexposed group with the four occupational exposure subgroups of GSH and GSSG (a, b, c, d; *p* = 0.000), and for the GSH / GSSG ratio (a, b, c, d; *p* = 0.710)

Furthermore, a scatter plot of data for the not exposed group and four sub-groups of exposures showed that the not exposed group had consistently higher values for GSH and GSSG (average of 1.58 μmMol / mL and 0.5221 μmMol / mL, respectively), while the different subgroups showed lower values with less variability which implied significant differences in GSH and GSSG (*p* = 0.000; Fig. 2). GSH and GSSG results for subgroups A, B, C, D shown nearly values like a same point in contrast with scale versus not exposed group (Fig. 2). In other hand, GSH/GSSG ratio show higher variability for the not exposed group and four sub-groups of exposures without significant differences (*p* = 0.710; Fig. 2).

### Effect of pesticide exposure on carbonyl levels and NO metabolites

**Fig. 3:**
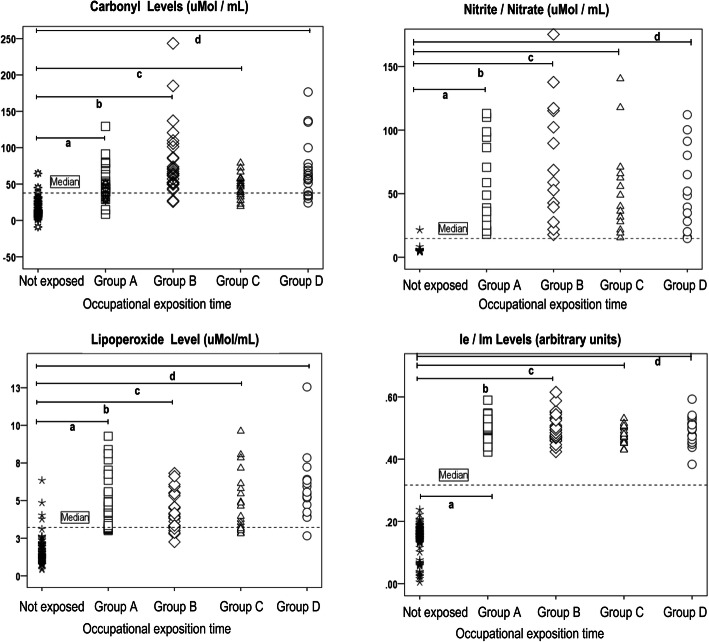
Carbonyl levels, nitrate-nitrite metabolites, LPO, and membrane fluidity levels in groups of exposed to OPs and not exposed group. The *p* values were obtained with a one-way ANOVA test at 95% confidence level. The significance value for the comparison between the unexposed group with the four occupational exposure subgroups A, B, C and D (a, b, c, d *p* = 0.000) is show

There were statistically significant differences in carbonyl levels and NO metabolites between the not-exposed group and the 4 occupational exposure subgroups (*p* = 0.000). Moreover, there were significant differences in carbonyl levels when sub-group A was compared with sub-groups B and D (*p* = 0.000 and 0.054, respectively), but not when sub-group A was compared with sub-group C (*p* = 0.999). Similarly, differences were found between sub-groups B and C (*p* = 0.000), and between sub-groups C and D (*p* = 0.041), but not between sub-groups B and D (*p* = 0.778). These results suggest that sub-groups B and D have higher values of carbonyl groups than sub-groups A and C (Fig. 3). Similarly, there were significant differences in NO metabolites of the four subgroups, when compared to not exposed group (*p* = 0.000), but not among the different sub-groups of exposed farmers. A plot of the distribution of carbonyl data for the not-exposed group and the 4 sub-groups of exposures revealed that the not-exposed group had lower values (mean = 13.5767 μmoles / mL), while the 4 sub-groups showed greater variability and higher values (range of mean = 47.51 to 80.05 μmoles / mL; *p* = 0.000; Fig. 3). The not exposed group had a low mean level of NO metabolites (5.0627 μmoles / mL), while the 4 subgroups had higher values (mean = 45.178 to 63.015 μmoles / mL; Fig. 3).

### Effect of pesticide exposure on lipid peroxidation (LPO) and membrane fluidity (MF)

There were statistically significant differences in LPO and MF between the not-exposed group and the 4-occupational exposure sub-groups (*p* = 0.000). These significant differences may be attributed to the not-exposure group, since when sub-group A was compared against sub-group B, C or D, there were no significant differences (*p* = 0.346, *p* = 1.000 and *p* = 0.288, respectively), nor were there significant differences when sub-group B was compared with sub-group C (*p* = 0.534), or sub-group C against sub-group D (*p* = 0.242), but when sub-group B was compared with sub-group D, there was significant difference (*p* = 0.003). In the plot of LPO data distribution for the not-exposed group and the 4 subgroups, the former showed lower values (mean = 1.5999 μmol / mL), when compared to the exposure sub-groups (4.2244 to 5.8014 μmol / mL), indicating statistical difference (*p* = 0.000; Fig. 3). For MF (Ie / Im), the not-exposed group had lower values (mean = 0.1453), but sub-groups A, B, C and D had much higher values (mean ranging from 0.4782 to 0.4975) (*p* = 0.000; Fig. 3). There were no significant changes in the 4 sub-groups.

## Discussion

### The most commonly used pesticides in La Cienega region

The significant differences in levels of oxidative stress markers between the occupationally exposed and not exposed subjects, and between the not-exposed group and the 4 exposure sub-groups may be due to the imbalance in the oxidant/antioxidant system. Participants in this study had chronic exposure mainly to OPs, carbamates, glyphosate and paraquat, wich they handled with minimal protection with a high dermal and inhalation exposure mainly. These findings are alarming because the region lacks toxicological studies that reveal the impact on farmer’s health. In addition, carbamate [23, 34, 35] and OPs [24, 27, 36] exposure increases levels of oxidative stress markers in both murine and human models. In different agricultural areas of Mexico the use of highly hazardous pesticides had negative effects on farm workers and their families, especially children [37, 38]. The General Bureau of Epidemiology, Ministry of Health, Jalisco, Mexico, has stated that between 2014 and 2018, there were approximately 76 cases of pesticide poisoning per annum in La Cienega, Jalisco area. This is equivalent to pesticide poisoning every 5 days. In Jalisco, México; the municipalities most adversely affected were La Barca (162 cases), Jocotepec (77 cases), Ocotlán (50 cases), Atotonilco el Alto (19 cases), and Tototlán (18 cases).

### Effect of pesticides on glutathione redox system

Significant differences were found in GSH and GSSG levels between the not exposed group and 4 occupational exposure sub-groups, indicating oxidative stress. A decrease in GSH/GSSG was observed as a result of exposure to OPs, carbamates and paraquat [7, 9, 23, 24, 27, 39–41]. However, unlike our results, studies that have analyzed GSH and GSSG specifically in erythrocytes after exposure to OPs (as was done in the present study) reported high levels [42, 43]. Particularly, differences in the GSH and GSSG responses depend not only on the type of sample analyzed, but also the type of OPs involved, and the duration of exposure [42].

The higher values to GSH and GSSG in the not exposed group, suggests that exposure to pesticides (regardless of the duration in years of use) affects human health, since the only group that maintained its functional antioxidant activity was not exposed group. On the other hand, the low levels of GSH and GSSG markers in exposed subgroups may be due to the low antioxidant defense induced by chronic exposure to pesticides, as reported by Spodniewska et al. [42] and Georgiadis et al. [43]. Conversely, other studies show that pesticide exposure may increase the GR activity, which converts GSSG to GSH, as an adaptation of the organism to prevent permanent oxidative damage [26, 34, 41]. However, our study showed that the levels of GSH and GSSG decrease significantly despite the previous evidence shown. Therefore, the observed decrease in both GSH and GSSG could be more important than the GSH/GSSG ratio for very low values.

### Effect of pesticides on carbonyl levels and NO metabolites

Sub-groups B and D had higher levels of carbonyls, when compared to sub-groups A and C. This may be due to oxidative deamination in response to the increased oxidative stress [16, 44, 45]. It was observed that the main pesticides to which sub-groups B and D were exposed were terbufos and carbofurans (OPs and carbamate, respectively; Fig. 1). Studies have demonstrated that exposure of porcine oocytes to 750–1000 μM of malathion (OPs) for 44 h increased protein oxidation and levels of carbonyls concentration [44]. In another study, exposure of rats to dichlorvos (OPs) at a dose of 47 mg/kg led to increases in carbonyls up to 95% higher than those of not exposed group [45]. Carbamate (carbofuran) exposure produces changes in carbonyls similar to those of OPs exposure, as revealed when young rats were exposed to these substances [46]. In this regard, Cattelan et al. have reported a significant increase in carbonyls in farmers exposed to carbamate-type pesticides [26]. The previous evidence is consistent with the results presented in Table 3, where a 4.5-fold increase in the formation of carbonyl groups of subjects exposed to pesticides versus subjects without occupational exposure is revealed (*p* = 0.000). Similarly, for NO metabolites, significant differences were observed among the 4 sub-groups, relative to the not exposed group, but not between the different exposure sub-groups. It is known that an increase in LPO levels goes together with an increase in NO metabolites [47]. The results of this study showed significant increases in NO_2_^−^/ NO_3_^−^ levels. These results agree with those obtained in previous studies on OPs (diazinon and chlorpyrifos) in which increases in nitrate/nitrite levels were reported in rats [48, 49]. Regarding occupational exposure to carbamates, studies have demonstrated that carbamate induces nitric oxide synthase, which results in increases in the levels of NO_3_^−^ and NO_2_^−^, along with enhancement of lipid peroxidation, and increases in levels of protein carbonyls groups [9, 50, 51] as seen in our results.

### Effect of pesticides on LPO and MF

The increase in LPO in the 4-occupational exposure sub-groups, especially in sub-group D relative to sub-group B may be related to increases in MDA and 4-HNE values with a longer exposure time to pesticides. This may be due to the fact that increased LPO levels were particularly associated with frequency of occupational exposure of terbufos (18.52 and 29.26%, B and D groups respectively) and carbamates (24.7 and 29.27%, B and D groups respectively) as shown in Fig. 1. The increase in LPO may be a response to the cholinergic hyperactivity which increased ROS and RNS production by both pesticides [21, 22, 50]. Similarly, exposure to pesticides generates H_2_O_2_ and OH^−^ radicals which produce peroxylipid radical (LOO·), and lipid-alkoxyl (LO·) radical which, on cyclization and reduction, give rise to MDA, 4-HNE and acrolein [16]. The increase in LPO with respect to the not exposed group is consistent with the reports of various researchers [27, 34–36].

Moreover, terbufos and carbamates are lipophilic molecules with high affinity for the plasma membrane [52], thereby inducing increased peroxidation [21, 22, 44, 51] and decreased membrane fluidity [21]. Studies by Rai et al. [51], Dhouib et al. [9], and Liu et al. [23], reported that exposure to carbamate increased levels of ROS and RNS, and decreased membrane fluidity. However, in this study, results showed a three-fold increase in membrane fluidity over the not exposed group, while among the 4 sub-groups, there were no significant variations in membrane fluidity. These data of membrane fluidity were unexpected. Interestingly, the effect of some insecticides on membrane fluidity depends on the cholesterol content [53]. Thus, a quantitative analysis of membrane lipids should be necessary to define this effect.

## Conclusion

La Cienega, Jalisco, Mexico is an important agricultural zone because of the economic impulse for the country. So, the use of pesticides is a common practice. In our results, the main pesticides used in the region are OPs, carbamates, glyphosate and paraquat with a high dermal and inhalation exposure, mainly. Particularly, exposure to OPs and carbamates shown an increase in levels of carbonyl groups, NO_2_^−^/ NO_3_^−^, lipoperoxidation and high mitochondrial membrane fluidity, as well as a decrease in the concentration of GSH and GSSG in exposed farmers versus the not exposed group. When evaluating groups A, B, C and D (intragroups) by years of exposure, a slight increase in oxidative markers and membrane fluidity were observed in those with more than 21 years of pesticide use (groups C and D), although it should be noted that it was not statistically significant analysis between groups. The increase in the oxidation levels that are found in farmers with occupational exposure constitute a theoretical basis on which to explain the high prevalence in the Cienega, Jalisco for neurodegenerative disease and cancer projected in future studies.

## Data Availability

The databases used during the current study are available from the corresponding author on reasonable request.

## Abbreviations

ROS: Reactive oxidant species
GR: Glutathione reductase
GSSG: Oxidized glutathione
GSH: Reduced glutathione
GPx: Glutathione peroxidase
OPs: Organophosphorus
H_2_O_2_: Hydrogen peroxide
OH^−^: Hydroxyl radical
RNS: Reactive nitrogen species
O_2_^−^: Superoxide anion
NO: Nitric oxide
ONOO^−^: Peroxynitrite
NO_2_^−^: Nitrite
NO_3_^−^: Nitrate
PUFAs: Polyunsaturated fatty acids
MDA: Malondialdehyde
4-HNE: 4-hydroxyalkene
AChE: Enzyme acetylcholinesterase
NMDA: N-methyl-D-aspartate
EDTA: Ethylene diamine tetra acetic acid
NADPH: Nicotinamide Adenine Dinucleotide Phosphate
DiPP: 1,3 dipyrylpropane
LPO: Lipid peroxidation
MF: Membrane fluidity
LOO: Peroxylipid radical
LO: Lipid-alkoxyl
SFAs: Saturated free fatty acids
